# The role of polygenic risk scores in breast cancer risk perception and decision-making

**DOI:** 10.1007/s12687-023-00655-x

**Published:** 2023-06-13

**Authors:** Leslie Riddle, Galen Joseph, Mikaella Caruncho, Barbara Ann Koenig, Jennifer Elyse James

**Affiliations:** 1 Department of Humanities and Social Sciences, University of California, San Francisco, San Francisco, CA USA; 2 Institute for Health and Aging, University of California, San Francisco, San Francisco, CA USA

**Keywords:** Polygenic risk score, Breast cancer, Precision medicine, Screening decision-making, Risk assessment, Population screening

## Abstract

Polygenic risk scores (PRS) have the potential to improve the accuracy of clinical risk assessments, yet questions about their clinical validity and readiness for clinical implementation persist. Understanding how individuals integrate and act on the information provided by PRS is critical for their effective integration into routine clinical care, yet few studies have examined how individuals respond to the receipt of polygenic risk information. We conducted an embedded Ethical, Legal, and Social Implications (ELSI) study to examine if and how unaffected participants in a US population breast cancer screening trial understood and utilized PRS, as part of a multifactorial risk score combining traditional risk factors with a genetic risk assessment, to make screening and risk-reduction decisions. Semi-structured qualitative interviews were conducted with 24 trial participants who were designated at elevated risk for breast cancer due to their combined risk score. Interviews were analyzed using a grounded theory approach. Participants understood PRS conceptually and accepted it as one of many risk factors to consider, yet the value and meaning they ascribed to this risk estimate varied. Most participants reported financial and insurance barriers to enhanced screening with MRI and were not interested in taking risk-reducing medications. These findings contribute to our understanding of how PRS may be best translated from research to clinical care. Furthermore, they illuminate ethical concerns about identifying risk and making recommendations based on polygenic risk in a population screening context where many may have trouble accessing appropriate care.

## Introduction

Polygenic risk scores (PRS) are being developed for a range of conditions, including breast and prostate cancers, cardiovascular disease, type 2 diabetes, Alzheimer’s disease, and several psychiatric disorders (Khera et al. 2018; Lambert et al. 2019; Mars et al. 2020). In combination with high-risk genetic variants and environmental or behavioral risk factors, PRS has the potential to improve the accuracy of clinical risk assessments, and thus advance prevention and screening interventions in those at high risk, while reducing overscreening and overtreatment of those at lower risk. However, questions remain about their clinical validity and readiness for clinical implementation (Elliott et al. 2020; Lewis and Green 2021; Torkamani et al. 2018), as well as about equitable access to both testing and follow-up care (Suckiel et al. 2022). This is especially pertinent in unequal and fragmented healthcare systems, like in the USA, where access to screening, prevention, and routine care is not available to all.

Another concern is the overrepresentation of European-ancestry samples in genomic databases used to generate PRS (De La Vega and Bustamante 2018; Hughes et al. 2021). Despite ongoing efforts to diversify the populations included in these genome-wide association studies, research has demonstrated differential accuracy of PRS across populations, and thus the potential to reinforce or exacerbate existing healthcare inequities if PRS is implemented using current knowledge (Adeyemo et al. 2021; Evans et al. 2022; Janssens 2019; Lambert et al. 2019; Lewis and Vassos 2020; Torkamani et al. 2018). Despite these concerns, some PRS are already being offered for clinical use by genetic testing companies using CLIA-certified labs, e.g., Ambry and Myriad (Ambry Genetics 2020a, b; Myriad Genetics 2020a, b). Research is ongoing to establish clinical utility, improve equity and applicability for diverse populations, discover how polygenic risk relates to risk from germline variants and non-genetic factors, and establish best practices for clinical implementation (Adeyemo et al. 2021; Arvanitis and Cainzos-Achirica 2022; Hao et al. 2022; National Human Genome Research Institute 2021; Steinberg et al. 2022).

In breast cancer, among the conditions where PRS research is most advanced (Hughes et al. 2021; Mavaddat et al. 2019), three large ongoing clinical trials — Perspective I&I in Canada, MyPeBs in Europe, and WISDOM in the USA — are testing PRS in combination with non-genetic factors in an effort to resolve the years-long controversy about breast cancer screening frequency, and to establish a risk-based paradigm for breast cancer screening and prevention (Brooks et al. 2021; L. Esserman et al. 2021; Mavaddat et al. 2019; Roux et al. 2022; Shieh et al. 2017). Understanding how individuals value, make meaning of, and act on the information provided by PRS is critical for their effective integration into routine clinical care. To date, few studies have examined how individuals respond to the receipt of polygenic information for breast cancer risk (Forrest et al. 2019; Willis et al. 2021; Yanes et al. 2020; Young et al. 2018). More recent studies are examining whether PRS, as part of a risk assessment that combines genetic and non-genetic factors, influences health behaviors including lifestyle changes, screening, and medication use (Muse et al. 2021; Wallingford et al. 2022; Widén et al. 2022; Yanes et al. 2021).

Our study contributes to this growing body of literature by exploring how women make meaning of PRS as part of a multifactorial breast cancer risk assessment in the WISDOM trial. WISDOM (Women Informed to Screen Depending on Measures of Risk, (NCT02620852)) is a US-based pragmatic trial of breast cancer screening comparing the conventional standard of annual mammograms to a personalized, risk-based screening (RBS) approach, including PRS, with the aim to inform and resolve the ongoing policy debates about screening frequency (Esserman 2017; Shieh et al. 2017). Starting in 2016, our team conducted a multimethod embedded Ethical, Legal, and Social Implications (ELSI) study of the WISDOM trial in which we examined how PRS was understood and utilized by participants who the trial identified as at elevated risk.

### The WISDOM trial

For participants in the RBS arm, WISDOM generates a five-year breast cancer risk score and recommends screening schedules accordingly. The WISDOM risk score is produced by combining two separate scores: the Breast Cancer Surveillance Consortium (BCSC) clinical risk prediction model, which includes breast density, family history, prior biopsies, age, and race/ethnicity (BCSC Breast Cancer Risk Calculator n.d.) and a PRS developed by WISDOM representing the effects of multiple single-nucleotide polymorphisms (SNPs) (Shieh et al. 2017). Participants also receive genetic testing for nine high and moderate penetrance genes (BRCA1, BRCA2, TP53, STK11, PTEN, CDH1, ATM, PALB2, and CHEK2) (Shieh et al. 2017); the WISDOM risk score is only used for those who test negative on the nine-gene panel. An online study, WISDOM is open to participants from anywhere in the USA aged 40–74 who have not had breast cancer.

**Fig. 1:**
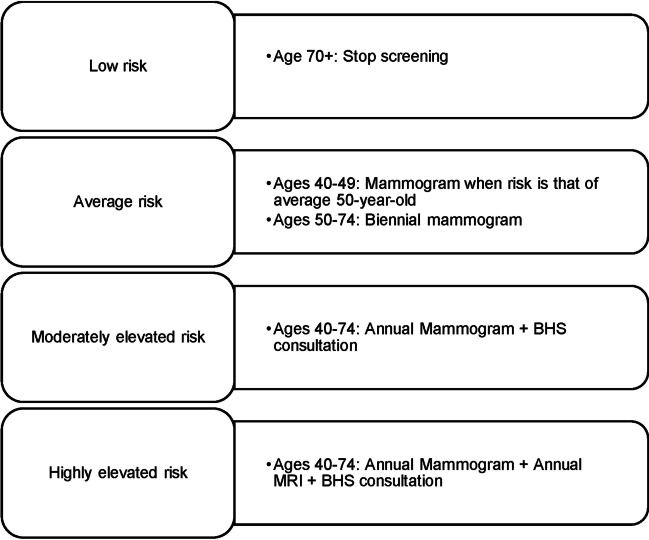
WISDOM screening recommendations

Participants whom WISDOM designates at moderately or highly elevated risk (see Fig. 1) due to factors other than pathogenic variants on the nine-gene panel receive a copy of their negative results report from Color Genomics and a WISDOM letter with their screening recommendation in their participant portal (Esserman 2017). The WISDOM letter explains the factors included in the risk assessment and offers a consultation with a WISDOM “Breast Health Specialist” (BHS), who is a physician, nurse, or genetic counselor, to discuss their risk assessment and risk-reducing medication.

As a pragmatic trial that aims to reflect the “real world,” WISDOM does not order or cover the cost of the screening and risk-reduction interventions that it recommends; participants are expected to confer with their providers about their screening schedule (see Fig. 1) and the use of risk-reducing medications (e.g., tamoxifen, raloxifene). As an adaptive trial, WISDOM incorporates new scientific advancements, including adding SNPs to the PRS calculation as new evidence is developed over the course of the trial. Screening recommendations may change due to these updates to WISDOM’s risk algorithm or due to updates to the participant’s annual questionnaire responses. The trial design has also evolved in response to unanticipated scenarios. For example, WISDOM originally planned to provide participants with their germline results, but did not anticipate disclosing PRS to participants. However, once WISDOM began telephone consultations to discuss screening and risk-reduction recommendations, it became clear that participants needed letters of medical necessity to share with their providers and insurance companies to access the additional recommended screening (i.e., MRI) and to justify the recommendation of risk-reducing medication. As a result, WISDOM began to return PRS directly to participants. Furthermore, in the WISDOM trial, the role of PRS in the determination of screening recommendations has changed due to factors such as presumptions about payor coverage or likelihood of uptake of risk-reduction recommendations, as well as emerging scientific knowledge.

## Methods

As part of our embedded ELSI study of WISDOM, our team of ethnographers observed hundreds of meetings of more than 20 ongoing WISDOM working groups which meet regularly to implement various aspects of the study (e.g., risk thresholds, statistical methods, and return of results), and interviewed key informants (e.g., study clinicians, investigators, and Breast Health Specialists). To elucidate the experience of receiving genomic risk information and personalized screening recommendations in the context of the trial, we invited eligible WISDOM participants to an interview by email. We interviewed WISDOM participants selected to include those at every risk level, every screening recommendation, and to oversample for socioeconomic diversity (race/ethnicity, insurance type, education level) to the extent possible within the limits of the WISDOM population. We also audio recorded telephone BHS consultations (average 35 min long) which were routinely conducted for participants designated at elevated risk for breast cancer. The combination of ethnographic observations, interviews, and audio recordings provided us with a multifaceted understanding of the WISDOM trial implementation. This paper focuses primarily on data from WISDOM participant interviews; data from our ethnographic observations are reported elsewhere (James and Joseph 2022).

Twenty-four of the WISDOM participants we interviewed had negative gene panel test results but were designated as elevated risk by WISDOM based on the combination of their BCSC score and PRS. Following WISDOM’s protocol, these 24 participants were recommended to screen either with annual mammography (moderately elevated risk; *n* = 12) based on being in the top 2.5th percentile of risk by age, or with alternating mammograms and breast MRI every six months (highly elevated risk; *n* = 12) based on having ≥ 6% five-year risk (the risk of an average BRCA2 carrier), and all were offered a consultation about risk-reducing medication. We conducted semi-structured qualitative interviews at two weeks post consultation (*n* = 24) and again six months later (*n* = 16). Six-month interviews were conducted with 11 highly elevated risk participants (one was lost to follow up) and five moderately elevated risk participants (two were lost to follow up, and based on analysis of the first five interviews, we determined that further six-month interviews with moderately elevated risk participants would not provide additional valuable data because these participants were not recommended to seek follow-up care during that time period). In the first interview, we sought to understand participants’ experience in the trial, their reactions to the information and intention to follow the recommendations provided by the study; in the second interview, we explored how participants had used that information to make decisions about screening and risk reduction. Interviews were conducted by phone between July 2017 and January 2021 by JJ, LR, GJ, and MC, masters and PhD-level researchers with expertise in the social sciences and qualitative research, until data saturation was reached. Interviews were audio recorded and lasted approximately 40–50 min. Interview guides included topics such as approach to health (including breast screening), family history of breast or other cancers, experience in WISDOM, understanding of genetic results and screening recommendations, breast cancer risk perception, and plans for follow up care. Demographic data were collected at the end of each initial interview (see Table 1). Pseudonyms were assigned to each participant in line with conventions of qualitative research in the social sciences and to ensure anonymity.

**Table 1:** Participant characteristics

Characteristic	*N*
Age	
40–49	4
50–59	9
60–69	9
70–74	2
Total	24
Race/ethnicity	
White	17
Hispanic	1
Multi-racial	4
Asian and Pacific Islander	2
Total	24
Screening recommendation	
Annual mammogram	12
Annual mammogram and annual MRI	12
Total	24
Education	
Some college	5
Associates degree	2
College degree	3
Advanced degree	14
Total	24
Annual household income	
< $25,000	1
$25,000–$49,999	1
$50,000–$74,999	3
$75,000–$100,000	2
> $100,000	17
Total	24
Health insurance	
Private	17
Public^a^	7
Total	24

^a^Four had Medicare, government funded health care for people aged 65 + ; of these, three had a supplemental private insurance policy. Two had Medicaid, government funded health care for people who have low income. One had Tricare, government funded health care for military personnel

The analysis presented here focuses on interviews and BHS recordings for these 24 participants. Interviews with participants who received positive germline results are reported elsewhere (James et al. 2022).

### Data analysis

Recordings of interviews and results disclosure sessions were transcribed and analyzed using standard techniques based on a grounded theory coding framework (Strauss and Corbin 1997) with data collection and analysis occurring concurrently. We used ATLAS.ti qualitative analysis software to enable searching and retrieval of coded text (ATLAS.ti 2022). Our research team worked collectively to code initial interview transcripts and develop a codebook. Subsequently, each transcript was coded by one member of the team and then reviewed by another to ensure consistent application of the codes; discrepancies were resolved through discussion and consensus. Transcripts and coded data were then discussed with the full research team to explore emerging themes and interpret the data. Coding and analysis for the six-month interviews and BHS recordings followed the same procedures with code lists refined and new codes added based on emerging findings in the follow-up interviews. We subsequently conducted focused coding to understand changes between the two interview time points for the identified themes and discussed these with the full team to refine the analysis.

## Findings

### Participants

The 24 interview participants ranged in age from 41 to 75, with a mean age of 58. The majority were white, had a household income above US $100,000 per year, had at least a college education, and were residents of California, reflecting the overall WISDOM study population at the time the interviews were conducted. The majority (18) had a family history of breast cancer; of these, 14 had a first degree relative with breast cancer. Most reported that prior to joining WISDOM, they had been having annual mammograms, and did not view the WISDOM recommendation of annual mammograms as a sign of elevated risk. All 24 had negative germline results but were classified at elevated risk due to their combined BCSC score and PRS. For 15 of the 24, PRS was the primary driver of WISDOM’s classification of them as elevated risk; they would not have been assessed to be at elevated risk based on BCSC alone. Other characteristics of these 24 participants are described in Table 1.

### Themes

Through our analysis, we elucidated how interview participants made meaning of PRS. We describe their meaning-making in relation to the following five issues: (1) understanding and acceptance of PRS; (2) breast cancer risk perception; (3) understanding of PRS as experimental; (4) decision-making about breast cancer screening; and (5) decision-making about risk-reducing medication.

#### Understanding and acceptance of PRS

Many participants were aware that they would be tested for a panel of breast cancer associated genes, but at the time they received their results, did not recall that a PRS would be part of their risk assessment. As a result, some expressed surprise when, despite testing negative for the nine breast cancer risk genes analyzed, they learned that they still had some genetic risk. As one participant said:

Well, I was surprised again because when I heard the main genetic ones were negative, I thought okay, that section’s clear. And so, I was a little bit surprised to hear that there were other factors that they were looking at. I mean, I didn’t mind that they were but…it was concerning to me when I got off the phone that oh, man, my risk went from what I thought was probably not significant at all, to, by the time you add all these things up, it is significant. (Jessie, 1st interview, highly elevated risk)

Despite not anticipating the PRS, participants accepted it as one of several risk factors that the WISDOM study took into account. Furthermore, most understood that SNPs are small genetic factors that increase their risk for breast cancer, using terms such as “multi genetic combinations,” “certain genetic material,” and “aberrations.” Participants also understood the risk associated with SNPs to be much lower than the risk associated with a BRCA variant, reflecting BHS explanations like this one:

So, it’s been found that there are some SNPs that are protective in terms of breast cancer. Some of them lower your risk, and there’s other SNPs that increase your risk. And when I say increase or decrease, it’s a fraction of a percentage. It’s nowhere near like the BRCA2 increase. This is just a very small increase. (BHS 1)

Several participants had a high scientific or medical literacy which informed their interpretation of PRS, such as one participant who worked in medical auditing and noted “reading a lot of science”; she demonstrated a strong grasp of how her polygenic risk compared to monogenic risk:

There are several genes that have variants that greatly increase the risk of breast cancer and I tested negative for all those variants. But there’s also a huge array of other genetic variations that can cause small increases in your risk. And unfortunately, I have inherited the high-risk variation on a large number of those small genetic factors. So, I’ve got a higher risk based on a whole bunch of small genetic changes. (Cheryl, 1st interview, highly elevated risk)

Only one participant expressed outright confusion, saying:

It was a lot to digest. She stayed on the phone with me like for 45 minutes. And…I said, “I don’t understand what that means, the genes and what they do with this particular test and how they fit,’” and I can’t even repeat what she said. It was way over my head. (Belinda, 1st interview, highly elevated risk)

When asked about the implications of their elevated polygenic risk for family members, participants had varied reactions, and often did not seem to have a clear understanding of whether or how SNPs might be inherited or shared among family members. A few reflected on the possibility that their family members could “have the same thing,” but were not sure. When asked how much of the SNPs are passed down, one participant responded:

I’m not sure, but I think with SNPs you have about a 50/50 chance on each one, but there’s so many of them. There were like maybe 200 in my evaluation. And so that’s not the same as the – I mean, the BRCA gene is 50/50 also but the BRCA gene has so much more impact. (Stacy, 1st interview, highly elevated risk)

#### Breast cancer risk perceptions

Despite understanding the concept of PRS, some participants left their BHS consultation somewhat confused about WISDOM’s overall assessment of their breast cancer risk, and several sought additional information to help interpret it. Other participants reported an understanding of the components of the WISDOM risk assessment — small genetic changes combined with other risk factors like family history and breast density — but if and how they integrated polygenic risk into their *personal* breast cancer risk assessment varied. For example, some participants considered PRS in relation to their understanding of themselves as healthy due to lifestyle behaviors which they assumed mitigated the risk assessed by WISDOM. One participant who was designated at highly elevated risk, and understood that her risk was higher than average, viewed her PRS in the context of her health more broadly, which made her feel she had an acceptable level of risk that did not require following WISDOM’s recommendation to screen more frequently and consider risk-reducing medication.

I feel like I’m in a good place in the fact that I have a healthy lifestyle. I eat healthy, I exercise, you know, I’m not obesely overweight, you know. So, I felt like, you know, given that, I would like to be below the general population, but I feel like you know what, 9%, eh, you know, better than 10%. (Nancy, 1st interview, highly elevated risk)

For some, the fact that the risk was spread across many less impactful risk factors in contrast to a monogenic germline variant like BRCA was also a comfort:

If I understood correctly, I don’t have a genetic risk for the big ones but that there is a combination of the polymorphisms that leaves me at a slightly increased risk, but it’s not just that alone. It’s a combination of other factors…It actually makes me feel more confident because it’s not just one thing; it’s a couple of things. And when there’s all these variables, I think that you have more of a chance of actually impacting it in a positive way than if it was just one thing like a BRAC [sic] gene or something and it’s sort of like oh, you’re doomed kind of thing. (Deb, 1st interview, moderately elevated risk)

Deb believed that changing her behavior such as exercising more and reducing her weight could lessen her risk. She felt more in control of managing her overall risk than she would have if she had learned of a pathogenic gene variant.

The distinction between polygenic and monogenic risk led some participants, upon learning they did not have a BRCA or other variant, to feel at lower risk than they had prior to joining WISDOM, despite their WISDOM assessment of elevated risk. For example, one participant described that learning she did not have a deleterious variant in one of the nine genes, which she had thought to be a possibility when she joined the study, changed her personal risk assessment:

I was really happy I don’t have any of the mutations that makes you get those really bad ones…That means my mom’s breast cancer was completely random and means my risks are probably a little bit lower than what I would’ve expected. I think, compared to another individual who had a mom who had BRCA1 mutation breast cancer, I definitely have a much lower risk than those individuals…I still have similar risk compared to the regular population, so I’m not worried too much. But, at the same time, I do feel I need to get routinely screened. (Lynn, 1st interview, highly elevated risk)

Many participants echoed the sentiment that while routine screening is important, they did not feel at acutely higher risk than the general population; while they had a higher five-year risk estimate than the average woman their age, that number did not always feel meaningful or actionable. For example, one participant reflected on what felt like an arbitrary distinction between elevated and not-elevated risk:

As I recall, she said the cutoff for recommending higher-risk frequent screening is 6.0% chance over the next year, next five years? I don’t remember. And that I had 6.3. So I thought that’s not a big difference, you know, from getting nothing extra to doing all this shit, you know...I hadn't asked her like, “What do you do for people at 6.1? What do you do with the people at 6.2, what do you do with people at 6.28?” It just felt a little arbitrary. (Angie, 2nd interview, highly elevated risk)

While risk exists along a continuum, clinical practice requires cut points for intervention or assessment that are, or can appear to be, arbitrary. This is necessary for prioritizing the allocation of resources in medicine, but may not feel meaningful to individuals attempting to make clinical decisions on the basis of these risk estimates. For Stacy, who described her risk as elevated compared to an average woman her age, her risk did not seem particularly actionable:

I do have what they’re calling SNPs, which put me up to a…7.2% chance of getting breast cancer, which is elevated for my age. I think for my age it’s more like a what, 2% or something or 3% maybe by now, ’cause I am getting older…I wasn’t at all surprised to find out that I’m somewhat elevated, just ’cause of all the time I’ve spent getting this looked at in the past…7.2% is not terrifying. If it had been 50%, that would be – I’d have to jump up out of my chair and do something about it. 7.2% is not shocking. (Stacy, 1st interview, highly elevated risk)

Stacy was not alone in describing 50% as the cut-off point at which she would have great concern. This perhaps speaks to challenges of conveying five-year risk estimates which will rarely if ever reach a level that sounds “shocking” but nonetheless are markedly higher than the risk of the general population.

#### Understanding of PRS as experimental

PRS continues to be updated over the course of the WISDOM trial, raising questions about if or how WISDOM participants understood PRS to be experimental or evolving and how that influenced their integration of PRS into both their own risk assessment and healthcare decision-making. Often, but not always, the novel or experimental nature of PRS was explained to participants by the BHS, one of whom noted it was “still under research.” Frequently, this came in the context of discussing how or why this risk assessment may be different from what the participant would receive as part of routine care. As one BHS described:

[SNPS are] not used clinically yet, but it’s all part of how medicine is striving towards becoming more personalized. And, unfortunately that data or that technique is not yet available in the clinical world and someday it will be, but that’s obviously information that you are getting from the WISDOM study, so I understand how that is kind of shocking. (BHS 2)

Most participants understood that the science behind PRS was evolving and trusted the study to provide the best risk assessment based on available evidence. As one participant put it, “they’re all just learning, you know.”

Cheryl, the woman who described reading a lot about science, responded as follows when asked about the use of PRS to inform screening decisions in WISDOM, given that it is not yet standard of care:

Oh, I think it’s fine because that’s the whole point, you’re trying to figure out whether the analysis of the polygenetic risk is actually helpful or not. And so you’ll be able to analyze people with similar genetic profiles, the ones who went through the screening and the ones who didn’t and compare their occurrence and time of identification and ultimate outcome to decide whether that’s actually a valid screening protocol or not that will actually influence population health. So I think it’s valid to do a study on it, an evolving field. (Cheryl, 2nd interview, highly elevated risk)

Lynn, who worked in the field of genomics, was asked if she had a sense of why her risk might have changed over the course of the study, resulting in a less aggressive screening recommendation now compared to the previous year. She spoke to the evolution of genetics research and was unsurprised that the models would change over time:

Probably their calculation has changed. Like I said, this is ongoing research of knowing what’s the risk for breast cancer, especially at the genetics part…so I’m not surprised they adjust this model from time to time. Maybe next year I’ll have a different risk score. I’m very open minded. I think the more data or the more research we have, we’ll have a better model all the time…I’d rather follow their recommendations, because I know if they make any change of their model it must be based on substantial evidence and new results that’s dependable. (Lynn, 1st interview, highly elevated risk)

However, she acknowledged that the current model may not account for all genetic risk, saying:

It’s not 100% clear whether I truly have a higher risk than the normal population, [whether there are] other SNPs that [could be] used to calculate the polygenic risk score, or another region that’s not even included in that formulation, right? (Lynn, 1st interview, highly elevated risk)

Dora, a researcher with expertise in genomics, noted feeling less “confident” about the use of PRS to guide her care. She asked the BHS whether PRS risk changes over time and was told there was not much data to determine how it affects people over the lifespan.

At the end of the day, I participated in the study, and now I don’t know what to do. But that’s because of the science…I am not as confident of the specificity of the results. I trust the results, that polygenic risk is real. I wish I knew more about the specifics within that, like I said. Is it on certain chromosomes? Is it certain mononucleotides? Is it in combination with certain other factors? … To say it’s polygenic risk is a pretty early, crude form of our understanding. It could be that it’s really just certain ones in combination with each other. And I have those or I don’t. (Dora, 2nd interview, highly elevated risk)

Dora is highlighting how the integration of PRS into clinical care is still an emerging science, raising questions for patients and providers about how best to apply this genomic knowledge to decisions about screening and risk reduction.

#### Decision-making about breast cancer screening

Several factors contributed to participants’ screening decision-making, especially about whether to follow the WISDOM recommendation to screen annually with mammogram and MRI. The most common considerations were insurance coverage and other practical barriers; whether their level of risk warranted additional screening with MRI; and the risks associated with MRI.

When we first interviewed participants, several indicated that they were willing to have an MRI but did not consider it urgent. WISDOM participants tend to have high household income with the vast majority having insurance coverage. Yet, the cost of MRI was a significant barrier for many participants we interviewed; even those with insurance were concerned about large co-pays, deductibles, or rejected claims. The experimental nature of PRS means that insurance companies may not see the MRI as medically necessary based on their own risk assessment of the patient. For example, Angie had some concerns about introducing MRIs into her screening regimen, but was willing to if the cost would be fully covered:

I won’t pay for it out of my pocket, and that’s the thing…none of these insurers will tell you if they’re going to cover it. But if I could get preauthorization, I guess I don’t mind. I don’t like a lot of radiation but it would be interesting to maybe get one or two and see what it shows compared to the mammogram. (Angie, 1st interview, highly elevated risk)

Other participants noted that this additional screening felt inconvenient and unnecessary:

I think they were asking for yearly mammogram plus MRI. Yeah. I think that was a little bit too much, really excessive…It’s hard for me to find time to see the doctor once a year. I just don’t want — you know, having unnecessary appointments. The transportation and the parking sometimes can prevent me from doing things like that. (Lynn, 1st interview, highly elevated risk)

For many participants, while annual mammograms felt like a necessary part of their routine preventative care, adding MRIs felt like, as one put it, “dessert”; nice to have but not essential. Another participant was unsure if her level of risk warranted an MRI even though she was aware that it might be a more effective screening tool than mammography for people like her with higher breast density. Nevertheless, she told us, “It depends on if my insurance will pay for the MRI.”

While some participants reflected on the benefits of an MRI, others were wary of potential risks, such as reactions to the contrast agent. For example, Cheryl felt that it would be more worthwhile to reserve the MRI for diagnostic use rather than screening:

First of all, I have a phobia about that kind of stuff, but also, when I read up about it, they said there is a fair number of people who get provoked into allergies to it. About one in a hundred applications have allergic reactions. And I actually get allergic to things a lot so I counted my chances higher than one in a hundred…I inquired about doing it without the gadolinium and they said it was much less useful because the gadolinium is really important to really see the smaller breast cancers when they start to grow and to really distinguish the cancerous tissue…So I sort of decided to reserve taking that risk for if I needed it for – more like if they actually thought they saw a cancer and they needed to define its boundaries. (Cheryl, 2nd interview, highly elevated risk)

Among those who had been considering the recommendation to have an MRI at the time of the first interview, several had scheduled or undergone an MRI at the time of the second interview. Some were still considering MRIs; others had decided against it. For example, Sandra felt her regular mammograms gave her enough assurance, and noted that at age 40, she was not in a position to take on additional screening: “Just weighing the finance aspect of it and then also, you know, my age, I feel comfortable not paying for it right now.” (Sandra, 2nd interview, highly elevated risk).

#### Decision-making about risk-reducing medication

In addition to screening recommendations, the BHS explained the potential benefits and risks of taking risk-reducing medications such as tamoxifen or raloxifene. We observed that many participants responded with skepticism, with several expressing that they would discuss the topic with their PCP. However, at the time of the first interview, two participants stated that they had started or planned to pursue medication for risk reduction. One was familiar with tamoxifen use for breast cancer treatment because her mother had taken it following a cancer diagnosis. She described her decision to start the medication, explaining:

I wanted to try it because I want to decrease my chances as much as possible. And one of the people that I talked to, they said that having a total hysterectomy, that I cut my chances in half already. I’m like okay, that’s good. And so, however I can get those odds down, I want to do it. I don’t want to have cancer, at all, ever. And so whatever I can do to facilitate that, that’s what I’m doing. (Tonya, 1st interview, moderately elevated risk)

Another participant who frequently had abnormal mammograms due to her breast density planned to request the medication from her doctor, but noted that she may not be considered high enough risk to get a prescription:

I talked to my mom about that and my mom even said that that would be, for someone like me, probably something good to do, just because of my own health history…[but] I don’t know that my risk would be high enough for them to prescribe me that. I would be willing to do it, especially if they were going to – somebody was going to keep track of that and to see where it goes. (Paula, 1st interview, moderately elevated risk)

At the time of the six-month interview, most participants continued to express reluctance to taking risk-reducing medication. Like Paula, many were unsure if their risk level warranted medication. Several participants expressed that the recommendation was “extreme” for what they had been told was a moderately elevated risk level. Others experienced the cognitive dissonance of feeling healthy yet being recommended to take a daily medication. Some of these participants were wary given that these medications are sometimes called chemoprevention, and are often prescribed to patients who have had breast cancer.

And the thing about what she said about the medication, is they really are cancer drugs, then if that’s really the case, then I would not want to do that. To take kind of toxic materials into my body when I don’t necessarily have cancer, that I know of. And so having, you know, a more enhanced screening process that to me seems to be less invasive would be the route that I’d prefer to take. (Deb, 2nd interview, moderately elevated risk)

A primary aversion to the idea of risk-reducing medication was the potential for side effects. While several medications were mentioned, for many participants, their prior knowledge or experience with tamoxifen influenced their decision-making:

I just feel like there’s risk with the tamoxifen, and I just am not willing to take those kinds of risks…hot flashes and increases your risk of endometrial cancer. I’m pretty sure that’s possible. With medication, you can decrease up to 50%, I think, [the risk] of ovarian cancer. But I feel like the risk of endometrial cancer is higher. It increases that risk. So, I just thought, why would I put myself in another risk category…Maybe if my risk were greater than nine percent over the five years or whatever. I just feel like the whole tamoxifen thing — I know of some people who have been on it and have had really a hard time with it. They had really bad migraines from it. (Nancy, 2nd interview, highly elevated risk)

For some participants, the potential side effects outweighed the potential risk reduction, especially for those who felt there were other less invasive methods of reducing risk, such as exercise and dietary changes. Several participants took pride in not taking any medications and wanted to maintain that if possible. As Stacy described:

Well, it’s a preventative, but I don’t really like to take meds. Like I’m not on any meds at all right now, and if I don’t have to take a med, I don’t like to take a med. Because I find that pretty much every med has some side effects…And so unless I was really strongly pushed towards it by either [my NP] or my doctor, I kind of feel that these six-month detailed check-ups are probably enough right now. If I had the BRCA gene, I might go on it. (Stacy, 2nd interview, highly elevated risk)

Because WISDOM does not prescribe medications, speaking with one’s doctor is both a necessary step in pursuing additional care and allows participants to ask additional questions or have a broader health history considered. However, several participants noted that they would need to see a specialist for risk-reducing medication because their PCP could not — or would not — provide it for them. As Deb went on to say:

I shared it ahead of time via email, and then I brought it up– you know, the recommendations about possibly taking that medication as a prophylactic. And she was pretty upset about it…She said she wasn’t going to do that. She said it was basically giving me cancer drugs when I don’t have cancer, and that if I wanted to do that, I could go see a specialist if I wanted to do something like that. But she wasn’t going to do it. She certainly recommended against it. (Deb, 2nd interview, moderately elevated risk)

A few participants were quite alarmed by the recommendation, with some questioning the validity of a risk assessment that would lead to suggesting medication. While rare, a few participants expressed distrust in the study or the pharmaceutical industry due to the recommendation. As one said, “I thought, is the manufacturer paying for this study? I didn’t want to make [the BHS] feel uncomfortable so I didn’t say that, but I thought ‘I’m not taking hormones to prevent hormonal cancer. Sorry.’” (Dora, 1st interview, highly elevated risk). Although the WISDOM study was not funded by pharmaceutical companies, this kind of distrust of the research enterprise and pharmaceutical companies is common.

## Discussion

Genetic and genomic screening is a key part of the promise of precision medicine: the right screening modality at the right time for each individual. The ongoing WISDOM trial seeks to test the hypothesis that screening based on a personalized risk assessment that integrates genetic and clinical risk factors will be as safe, less morbid, and have greater healthcare value than annual mammography (L. J. Esserman 2017). While that question will be answered in time, our embedded ELSI study examined if and how WISDOM participants understood and utilized PRS in the research setting to make screening and risk-reduction decisions as part of their clinical care.

As in other studies of how risk perception drives health decision-making (Holmberg et al. 2015), we found that participants integrated many factors into their personal risk assessment, including factors distinct from those included in WISDOM’s risk model such as lifestyle, family history beyond first degree relatives, and other health conditions. Furthermore, some participants gave these factors more weight than their PRS, which aligns with other recent research on receipt of PRS that found that individuals personalize their risk assessment based on various factors that are perceived as stronger drivers than PRS (Willis et al. 2021). While WISDOM participants accepted PRS as a valid risk factor to include as part of their overall risk assessment, they infrequently used it to guide their screening and risk-reduction behaviors in the context of other perceived risk factors. Like those in Willis and colleagues' study, their PRS did not lead to behavior change; while participants understood their risk to be higher than the “average” woman, most did not feel their risk estimate was high enough to pursue MRIs or take risk-reducing medications, a finding that is consistent with other studies demonstrating low uptake of chemoprevention medications among high-risk women (Flanagan et al. 2019; Ropka et al. 2010; Smith et al. 2016). For those participants who were interested in enhanced screening, financial and insurance barriers made an annual MRI feel like an “extra” rather than a necessary aspect of annual preventative care (like mammograms). Likewise, several participants interested in taking risk-reducing medication reported that their doctors were unable or unwilling to prescribe it.

Although WISDOM is a population screening trial, most of our interview participants reported a family history of breast cancer and thought of themselves as having higher risk than the general population when they joined the study. In fact, some joined the study to gain access to free BRCA testing because they believed they had a good chance of testing positive for a BRCA variant. Thus, finding out they were negative for BRCA reduced their perceived level of risk, despite being classified as elevated risk by WISDOM due to their PRS (which for some participants conferred the same five-year risk of breast cancer as a BRCA2 carrier). A high PRS was not viewed by participants as requiring immediate risk-reducing action or communication with family members like a pathogenic BRCA variant. In results disclosure discussions, the BHS typically contrasted BRCA with the cumulative risk of SNPs even while emphasizing that risk of cancer from a pathogenic BRCA variant is not 100%. Given how recently PRS has entered the clinical space, most research on the relationship between receiving positive genetic test results and decision-making about risk-reduction behaviors has focused on monogenic variants, such as those that cause hereditary cancer syndromes like HBOC and Lynch (Ladd et al. 2020; Steel et al. 2017). While certain social and ethical issues that arise with monogenic testing, such as relevance of results for family members, will continue to be applicable in the polygenic context (A. C. F. Lewis and Green 2021), our research highlights that polygenic risk may influence healthcare decision-making (and family communication about risk) quite differently and suggests that different approaches to clinical communication may be needed if screening and risk-reduction behavior change based on PRS is the goal. WISDOM has introduced a “breast health decisions tool” to standardize communication about risk and risk reduction with participants (Keane et al. 2021), but further research is needed to understand how the tool may affect participants’ risk perception and their screening and risk-reduction decisions. Similar research interventions have shown promise; for instance, one study found that returning risk information for cardiovascular disease using traditional risk factors and a PRS via an interactive web-based tool had a positive impact on health behavior (Widén et al. 2022).

The fact that WISDOM is a pragmatic clinical trial blurring the boundary between research and clinical care may be important to consider in interpreting the lack of urgency and healthcare barriers participants discussed. Participants received genetic risk information in a research setting and understood the experimental nature of its application to screening; nevertheless, they were asked to make screening and risk-reduction decisions in the clinical space in collaboration with providers who may or may not be familiar with the WISDOM trial or even with polygenic risk scores. Research has demonstrated that many primary care providers feel ill prepared to incorporate genomics into their routine practice (Haga et al. 2019; Sharma et al. 2021), with one study finding that PCPs were generally less enthusiastic about using PRS to make decisions about whether to prescribe medications and desired more training and evidence-based guidelines for care (A. C. F. Lewis et al. 2022). WISDOM provides real-world evidence of how individuals may weigh the benefits and costs of additional screening and risk reduction where health systems and insurance barriers play a key role, which raises questions about who will ultimately benefit from wide-scale population screening and personalized risk assessment. One recent qualitative study with racially, ethnically, and socioeconomically diverse adults in a US biobank explored participants’ perceived barriers to adoption of hypothetical PRS-related follow-up care and recommendations, identifying barriers such as inadequate insurance coverage and understanding of PRS, financial constraints, and limited healthcare access (Suckiel et al. 2022). Despite our highly resourced study participants, we found similar barriers in our study, which raises ethical concerns about identifying risk and making recommendations that may be out of reach for much of the US population. The US healthcare landscape is fragmented and unequal; genomic medicine, like other new technologies, has the potential to exacerbate disparities without attention to equitable implementation (Clarke and van El 2022; Jooma et al. 2019). Our study highlights the need for non-genetics provider knowledge and buy-in of PRS, including how to incorporate it into clinical (and personal) risk assessments to guide patient decision-making and inform recommendations for care. It also illuminates the role that insurance coverage will undoubtedly play in patient decision-making and access to recommended care. The still experimental nature of PRS means that it may be out of reach for those whose insurers do not recognize it as evidence-based care, and even more so, those who are uninsured.

### Limitations

This study has some limitations. It reflects a specific period of the WISDOM trial which has evolved since completing our interviews and is ongoing. Our participants represent the cohort enrolled in the first four years of the WISDOM trial, the majority of whom were white, high income, insured, and had higher levels of education and genetic/medical literacy. We were not able to sample from the more diverse cohort enrolled in subsequent years. In addition, WISDOM’s early cohorts, and thus our interview participants, were more likely to have a family history of breast cancer, and therefore may not represent a true population screening context.

## Conclusions

Breast cancer remains the most common cancer among women in the USA, demanding novel approaches to identify those at highest risk. Although clinical risk models have evolved to capture an increasing number of risk factors, patients often incorporate less “quantifiable” factors into their personal risk assessment, such as overall feelings about their health and family history and health behaviors like diet and exercise, highlighting the need for counseling approaches and risk models that take such factors into account (Schwartz et al. 2021). Furthermore, advances in population screening that identify those most at risk but do not exacerbate existing disparities are urgently needed. Cost and access issues created barriers for our interview participants, despite their relative wealth, education, and racial privilege. While they did not report high levels of concern about the risk conferred by their PRS, the barriers they faced raise questions about how PRS could or should be integrated into clinical risk assessments and whether all patients regardless of medical literacy, insurance status, and income level will be able to access appropriate screening and follow-up care based on risk assessments that rely on or incorporate a PRS.

A pragmatic trial like WISDOM demonstrates that translation and implementation of emerging genomic discoveries may be as complicated as the science itself; WISDOM is attempting to simultaneously understand and push forward the science of PRS and its application to breast cancer screening. What is experimental in this trial is not the role of PRS in breast cancer risk in and of itself, but the application of PRS to screening and risk-reduction decisions. While our findings describe the experiences of women participating in a research trial, their interest in and ability to modify screening or risk-reducing behavior is dependent on their real-world clinical environment. In the context of an unequal US healthcare system, only the most privileged participants may be able to decide whether or not to screen as recommended. Thus, our ELSI study of the WISDOM trial offers important insights into how PRSs are understood and acted upon in a population screening context, as well as insights into the practical challenges and ethical implications of translating PRS from research to clinical care.

## Data Availability

The participants of this study did not give written consent for their data to be shared publicly, so due to the potentially identifiable nature of data, supporting data is not available.
